# Platelet-associated parameters in patients with psoriasis: A PRISMA-compliant systematic review and meta-analysis

**DOI:** 10.1097/MD.0000000000028234

**Published:** 2021-12-17

**Authors:** Li Li, Jianxiu Yu, Zhongwei Zhou

**Affiliations:** a Department of Clinical Laboratory, Binhai County People's Hospital, Binhai, Jiangsu, China; b Department of Clinical Laboratory, Yancheng Third People's Hospital (The Affiliated Yancheng Hospital of Southeast University Medical College, The Sixth Affiliated Hospital of Nantong University, The Yancheng School of Clinical Medicine of Nanjing Medical University), Yancheng, Jiangsu, China.

**Keywords:** mean platelet volume, meta-analysis, platelet count, platelet distribution width, platelet-to-lymphocyte ratio, psoriasis

## Abstract

**Background:**

The relationship between platelet-associated parameters and psoriasis has been controversial. The purpose of our meta-analysis was to assess whether platelet count, platelet-to-lymphocyte ratio (PLR), mean platelet volume (MPV), and platelet distribution width (PDW) are associated with psoriasis.

**Methods:**

We performed a thorough documentation retrieval via PubMed, EMBASE, and Web of Science until June 2021. Pooled standardized mean differences (SMDs) and 95% confidence intervals (CIs) were calculated using a random-effects model.

**Results:**

Overall, 22 studies involving 1749 patients with psoriasis and 1538 healthy controls were selected for the meta-analysis. The outcomes showed that platelet count presented non-significant differences between psoriatic patients and normal individuals (SMD = 0.12, 95% CI =  −0.07 to 0.32, *P* = .210), while PLR (SMD = 0.28, 95% CI = 0.03–0.53, *P* = .031), MPV (SMD = 0.55, 95% CI = 0.30–0.79, *P* < .001), and PDW (SMD = 0.29, 95% CI = 0.03–0.55, *P* = .027) were remarkably greater in the psoriatic patients than in the healthy individuals, and similar results were found in subgroup analyses. The analytical results of susceptibility revealed that the outcomes were robust, and no evidence of substantial publication bias was identified.

**Conclusion:**

Patients with psoriasis present significantly higher PLR, MPV, and PDW than healthy individuals, suggesting that psoriasis is accompanied by low-grade systemic inflammation and platelet activation.

## 1 Introduction

Psoriasis is a chronic inflammatory disease of the skin characterized by erythema, papules, and scales, which affects approximately 2% to 3% of the general population.^[1]^ The disease mainly involves the skin; however, it is also associated with various comorbidities, such as arthritis, metabolic syndrome, and cardiovascular disease.^[2]^ Although the etiology and pathogenesis of psoriasis remain unclear, inflammation and immunity are involved in its development and progression.^[3,4]^

Recently, increasing evidence has suggested that platelets play a role in regulating immune and inflammatory responses beyond their traditional role as regulators of hemostasis and wound repair.^[5–7]^ Platelets are activated and bind to leukocytes in immune-mediated inflammatory responses, and platelet-leukocyte interactions release a high concentration of cytokines, chemokines, and growth factors.^[8,9]^ Psoriasis is characterized by leukocyte infiltration into the skin, which may arise from platelet activation and subsequent excessive cytokines and chemokines.^[10,11]^ Platelet activation can be assessed by changes in platelet count and volume, and mean platelet volume (MPV) and platelet distribution width (PDW) are good indicators of platelet size.^[12]^ Platelet-to-lymphocyte ratio (PLR) has recently been introduced as a superior marker to monitor systemic inflammation, which has been shown to be higher in a multitude of inflammatory disorders.^[13]^ An increased PLR may stem from an elevated platelet count (PLT) , decreased lymphocyte number, or both.

Over the past few years, the comparisons of different platelet-associated parameters between psoriatic patients and healthy individuals have been significantly inconsistent in different studies. Moreover, most studies evaluating the relationship between them have been limited by the small sample size. Therefore, in order to better understand the role played by different platelet-associated parameters in the development of psoriasis, we conducted a systematic review and meta-analysis of published studies that compared PLT, PLR, MPV, and PDW between psoriatic patients and healthy individuals.

## 2 Materials and methods

The present systematic review was performed according to the Preferred Reporting Items for Systematic Reviews and Meta-Analyses (PRISMA) guidelines.^[14]^ Since this study is a systematic review and meta-analysis, no ethical approval was required.

### 2.1 Search strategy

A thorough documentation retrieval was carried out using electronic databases including PubMed, EMBASE, and Web of Science until June 18, 2021. The retrieval keywords are presented below: (psoriasis OR psoria∗) AND (“platelet count” OR “platelet number” OR “platelet-to-lymphocyte ratio” OR “platelet-lymphocyte ratio” OR PLR OR “mean platelet volume” OR MPV OR “platelet distribution width” OR PDW). We searched the bibliography of the relevant literature for more related information.

### 2.2 Research selection

A research would be taken as appropriate if it reached the pre-determined selection standards below: studies in patients ≥18 years old; studies contrasting platelet-associated parameters (PLT, PLR, MPV, or PDW) between psoriatic patients and normal controls; and studies written in English. Studies were deemed unsuitable if no necessary data or comparative groups; sample repetition with other studies; or reviews, case presentations, mails, or conference summaries.

### 2.3 Data extraction

Data were obtained by 2 researchers separately (Li Li and Jianxiu Yu), and conflicts were resolved by another researcher (Zhongwei Zhou). The following data were extracted from each appropriate research: the name of the first author, publishing time, research regions, and research design; relevant demographic information and clinical status of psoriatic cases, including age, sex, disease duration, and the Psoriasis Area and Severity Index (PASI) score; specimen scale, PLT, or PLR, MPV, or PDW in psoriatic cases and healthy controls.

### 2.4 Quality evaluation

Two investigators (Li Li and Jianxiu Yu) assessed the quality of the included studies according to the Newcastle-Ottawa Quality Assessment Scale (NOS) for observational studies modified by van Dijk et al.^[15]^ As the maximum score stood at 9, the quality of the included articles was considered inferior, medium, or superior according to a score of 0 to 3, 4 to 6, or 7 to 9, separately.

### 2.5 Statistical analysis

Our team adopted Stata15.0 (StataCorp LP, TX) to carry out the entire study statistically. For each study, we calculated standardized mean differences (SMDs) and 95% confidence intervals (CIs) to estimate the diversity in platelet-associated parameters between psoriatic patients and healthy controls. A random-effects model (REM) was adopted for the estimate, which was more conserved than the fixed-effect model.^[16]^ The heterogeneous results of statistics among studies were evaluated using the chi-square test, and significant levels were *P* < .10. The qualification of heterogeneous levels was determined by *I*^2^, and *I*^2^ > 50% indicated significant heterogeneity. To unveil the reasons for the heterogeneous results, our team carried out subgroup studies and meta-regression analysis. The former was classified by the research region and research design. For meta-regression analysis, SMD was the dependent variable, and age, the percentage of males, sample size, disease duration, PASI score of psoriatic patients, and publishing time of included studies were chosen as independent variables. Sensitivity studies were carried out by sequentially reducing 1 study in each turn to evaluate the stability of the results. Publication bias was first evaluated by visually inspecting funnel plots and statistically assessed using Egger test.

The *P* score below .05 had significance on statistics, unless otherwise pointed out.

## 3 Results

### 3.1 Research selection

**Figure 1:**
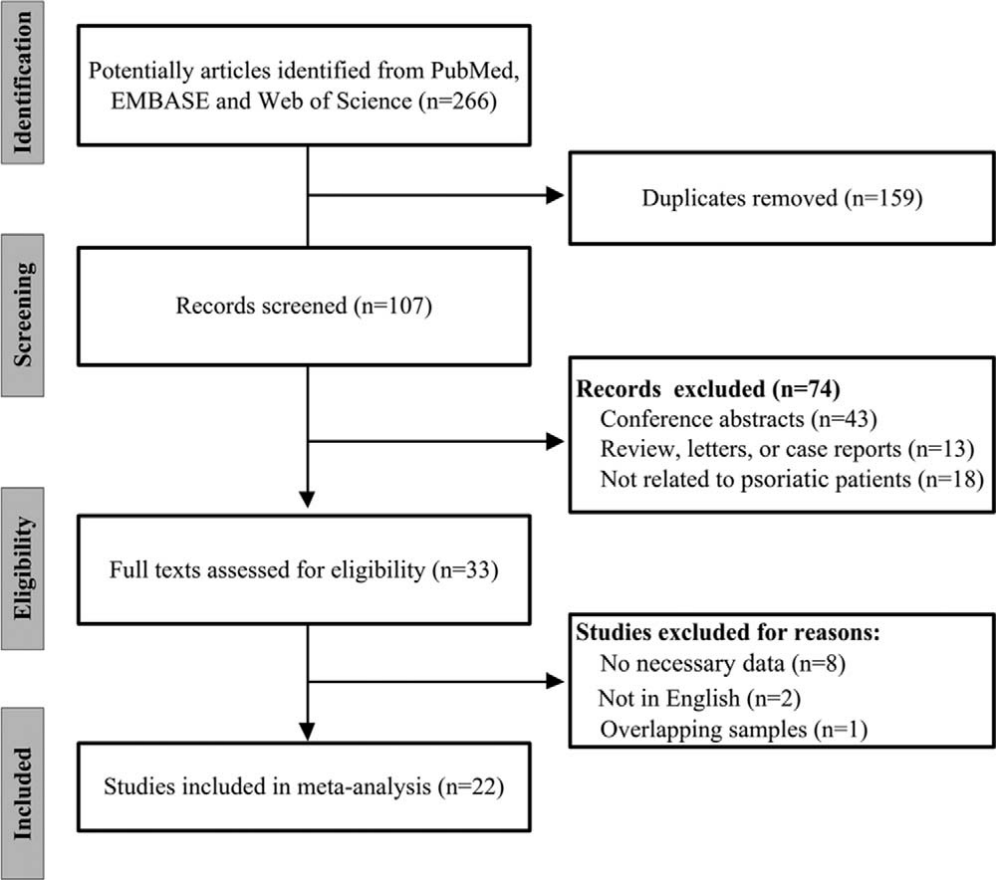
The process of selecting researches.

We initially identified 266 records; after the elimination of 159 duplicate publications, 107 records remained, of which 74 publications were excluded according to the title and abstract. Of the remaining 33 publications selected for full-text studies, 11 were discarded due to missing outcome data, non-English language, or repetitive information with other studies. Eventually, 22 articles were eligible for the meta-analysis,^[17–38]^ and the process of the selected and discarded studies is displayed in Figure 1.

### 3.2 Features of included researches

The primary characteristics of the selected studies are summarized in Table 1. The publications of the selected studies were from 2008 to 2020 and included 1749 psoriatic patients and 1538 healthy controls. Half of the studies (11/22) were cross-sectional, while the other half were case-control studies. Fourteen studies were carried out in Turkey, 2 in India, 2 in Egypt, 2 in Korea, and 2 in Japan and Pakistan. Twenty-one studies provided information on the age and sex ratio of the patients. Thirteen studies provided data on disease duration, and 20 studies reported data on the PASI scores of patients.

**Table 1:** Demographic and clinical data of psoriatic patients included in this meta-analysis.

References	Region	Study design	Age (yrs)	Gender (M/F)	Disease duration (yrs)	PASI score	Quality score
Karabudak, 2008^[17]^	Turkey	Cross-sectional	23 ± 4	20/0	4 ± 4	13 ± 7	7
Tamagawa-Mineoka, 2010^[18]^	Japan	Cross-sectional	47.0 ± 12.5	15/6	NA	16.2 ± 1.63	4
Canpolat, 2010^[19]^	Turkey	Cross-sectional	NA	31/27	13.5 ± 4	10.7 ± 7	5
Saleh, 2013^[20]^	Egypt	Case-control	31.56 ± 10.09	13/12	6.22 ± 5.99	22.59 ± 18.07	6
Yurtdaş, 2014^[21]^	Turkey	Cross-sectional	39 ± 9	32/19	7 ± 5	16 ± 11	8
Ahmad, 2014^[22]^	Pakistan	Case-control	40.2 ± 10.4	16/14	NA	NA	4
Chandrashekar, 2015^[23]^	India	Cross-sectional	44.5 ± 12.89	52/10	1.70 ± 1.74	14.90 ± 9.83	9
Kim, 2015^[24]^	Korea	Case-control	39.82 ± 15.16	114/62	NA	8.56 ± 7.53	6
Pektas, 2016^[25]^	Turkey	Cross-sectional	42.17 ± 11.98	84/88	NA	7.75 ± 6.85	8
Işik, 2016^[26]^	Turkey	Case-control	46.00 ± 14.62	23/22	13.7 ± 11.3	16.3 ± 11.2	4
Unal, 2016^[27]^	Turkey	Cross-sectional	32.41 ± 18.03	19/78	NA	5.96 ± 0.95	5
Kim, 2016^[28]^	Korea	Cross-sectional	38.0 ± 16.6	62/49	NA	NA	5
Raghavan, 2017^[29]^	India	Case-control	46.10 ± 11.99	38/12	NA	15.88 ± 2.51	5
Kiliç, 2017^[30]^	Turkey	Case-control	37.66 ± 14.63	22/19	16.53 ± 11.98	12.48 ± 8.39	5
Polat, 2017^[31]^	Turkey	Case-control	36.58 ± 9.82	25/21	13.21 ± 8.24	9.08 ± 8.78	6
Korkmaz, 2018^[32]^	Turkey	Cross-sectional	43 ± 9.3	22/16	NA	3.04 ± 2.18	6
Farag, 2018^[33]^	Egypt	Case-control	41.47 ± 8.16	40/30	8.4 ± 3.4	12.85 ± 4.97	7
Yavuz, 2019^[34]^	Turkey	Cross-sectional	40.5 ± 15.2	NA	9.4 ± 7.3	8.1 ± 5.2	5
Yorulmaz, 2020^[35]^	Turkey	Case-control	43.60 ± 13.6	102/69	9.5 ± 8.89	6.5 ± 7.56	8
Aktaş, 2020^[36]^	Turkey	Case-control	38.28 ± 12.48	NA	10.0 ± 6.67	9.65 ± 7.78	4
Sirin, 2020^[37]^	Turkey	Cross-sectional	37.91 ± 13.46	31/29	9.14 ± 7.81	10.2 ± 9.56	8
Özkur, 2020^[38]^	Turkey	Case-control	45.4 ± 16.3	11/17	NA	5.5 ± 3.4	5

M/F = male/female, PASI = the Psoriasis Area and Severity Index, NA = not accessed.

The outcomes of the evaluation of qualities are also presented in Table 1. Seven studies were deemed as outstanding (NOS ≥ 7), whereas the remaining 15 were deemed to have medium excellence (4 ≤ NOS ≤ 6). There have been no studies with low-quality ratings.

### 3.3 Outcomes of platelet-associated parameters of patients and controls

The outcomes of platelet-associated parameters of psoriatic patients and healthy individuals are summarized in Table 2. The number of patients in the psoriatic and healthy groups changed from 20 to 320 and 20 to 200, respectively. Thirteen researches displayed that the PLT ranged from 202.0 to 296.8 × 10^3^/μL in the sufferers and 197.0 to 276.0 × 10^3^/μL in the healthy individuals. Seven studies reported PLR ranging from 101.3 to 159.2 in the patient group and 101.0 to 220.0 in the control group. Seventeen studies demonstrated that the MPV ranged from 8.0 to 13.49 FL in the patients and 7.0 to 10.46 FL in the healthy individuals. Five researches displayed that the PDW ranged from 11.48 to 16.8 FL in the sufferers and 10.98 to 16.77 FL in the healthy individuals.

**Table 2:** Results of platelet-associated parameters in psoriatic patients and healthy controls.

References	Patient group					Control group				
	Sample size (N)	PLT count (×10^3^/μL)	PLR	MPV (FL)	PDW (FL)	Sample size (N)	PLT count (×10^3^/μL)	PLR	MPV (FL)	PDW (FL)
Karabudak, 2008^[17]^	20	NA	NA	10.0 ± 1.0	NA	20	NA	NA	7.0 ± 1.0	NA
Tamagawa-Mineoka, 2010^[18]^	21	223.0 ± 44.3	NA	NA	NA	22	220.0 ± 34.3	220.0 ± 34.3	NA	NA
Canpolat, 2010^[19]^	58	NA	NA	8.0 ± 0.7	NA	95	NA	NA	7.3 ± 0.8	NA
Saleh, 2013^[20]^	25	NA	NA	9.16 ± 1.28	NA	25	NA	NA	9.96 ± 1.85	NA
Yurtdaş, 2014^[21]^	51	202 ± 95	124.0 ± 98.0	NA	NA	37	197 ± 69	101.0 ± 60.0	NA	NA
Ahmad, 2014^[22]^	30	NA	NA	8.24 ± 1.22	NA	30	NA	NA	7.29 ± 0.77	NA
Chandrashekar, 2015^[23]^	62	NA	NA	13.49 ± 2.10	13.14 ± 2.79	62	NA	NA	10.46 ± 1.70	11.39 ± 1.45
Kim, 2015^[24]^	176	248.0 ± 47.69	NA	9.92 ± 0.73	11.48 ± 1.45	101	249.9 ± 41.23	NA	9.72 ± 0.59	10.98 ± 1.16
Pektas, 2016^[25]^	172	295.12 ± 81.32	137.5 ± 61.3	10.59 ± 1.81	NA	128	239.4 ± 59.3	105.0 ± 45.7	9.72 ± 1.28	NA
Işik, 2016^[26]^	45	NA	NA	8.98 ± 1.14	NA	44	NA	NA	9.19 ± 1.28	NA
Unal, 2016^[27]^	320	277.7 ± 73.4	NA	8.25 ± 1.15	NA	200	265.1 ± 59.7	NA	7.44 ± 1.63	NA
Kim, 2016^[28]^	111	256.6 ± 60.1	140.7 ± 114.9	NA	NA	94	248.6 ± 38.9	132.3 ± 41.1	NA	NA
Raghavan, 2017^[29]^	50	220.4 ± 61.9	NA	9.65 ± 2.07	NA	50	256.4 ± 81.0	NA	8.51 ± 1.64	NA
Kiliç, 2017^[30]^	41	NA	NA	8.79 ± 0.86	NA	90	NA	NA	8.42 ± 0.74	NA
Polat, 2017^[31]^	46	296.8 ± 83.2	159.2 ± 64.2	9.43 ± 1.13	NA	46	264 ± 55.8	125.0 ± 38.1	9.60 ± 1.03	NA
Korkmaz, 2018^[32]^	38	274 ± 100.5	NA	10.2 ± 0.9	12.1 ± 1.8	35	263 ± 104	NA	10 ± 1.7	11.6 ± 2.7
Farag, 2018^[33]^	70	NA	NA	10.08 ± 1.07	NA	60	NA	NA	8.92 ± 0.78	NA
Yavuz, 2019^[34]^	60	260.0 ± 73.3	101.3 ± 35.6	8.90 ± 0.96	NA	30	268.2 ± 68.1	108.4 ± 47.2	8.80 ± 0.89	NA
Yorulmaz, 2020^[35]^	171	262.0 ± 62.8	128.9 ± 50.7	NA	12.2 ± 1.8	171	276.0 ± 79.6	103.7 ± 35.9	NA	12.1 ± 2.3
Aktaş, 2020^[36]^	94	NA	106.9 ± 38.4	NA	NA	118	NA	110.8 ± 37.7	NA	NA
Sirin, 2020^[37]^	60	249.8 ± 59.4	NA	8.71 ± 0.83	16.8 ± 0.43	50	248.2 ± 53.1	NA	8.63 ± 0.78	16.77 ± 0.42
Özkur, 2020^[38]^	28	291.5 ± 44.5	NA	8.9 ± 1.3	NA	30	265 ± 36.5	NA	8.2 ± 1.4	NA

PLT = platelet count, PLR = platelet-to-lymphocyte ratio, MPV = mean platelet volume, PDW = platelet distribution width, NA = not accessed.

### 3.4 Meta-analysis

**Figure 2:**
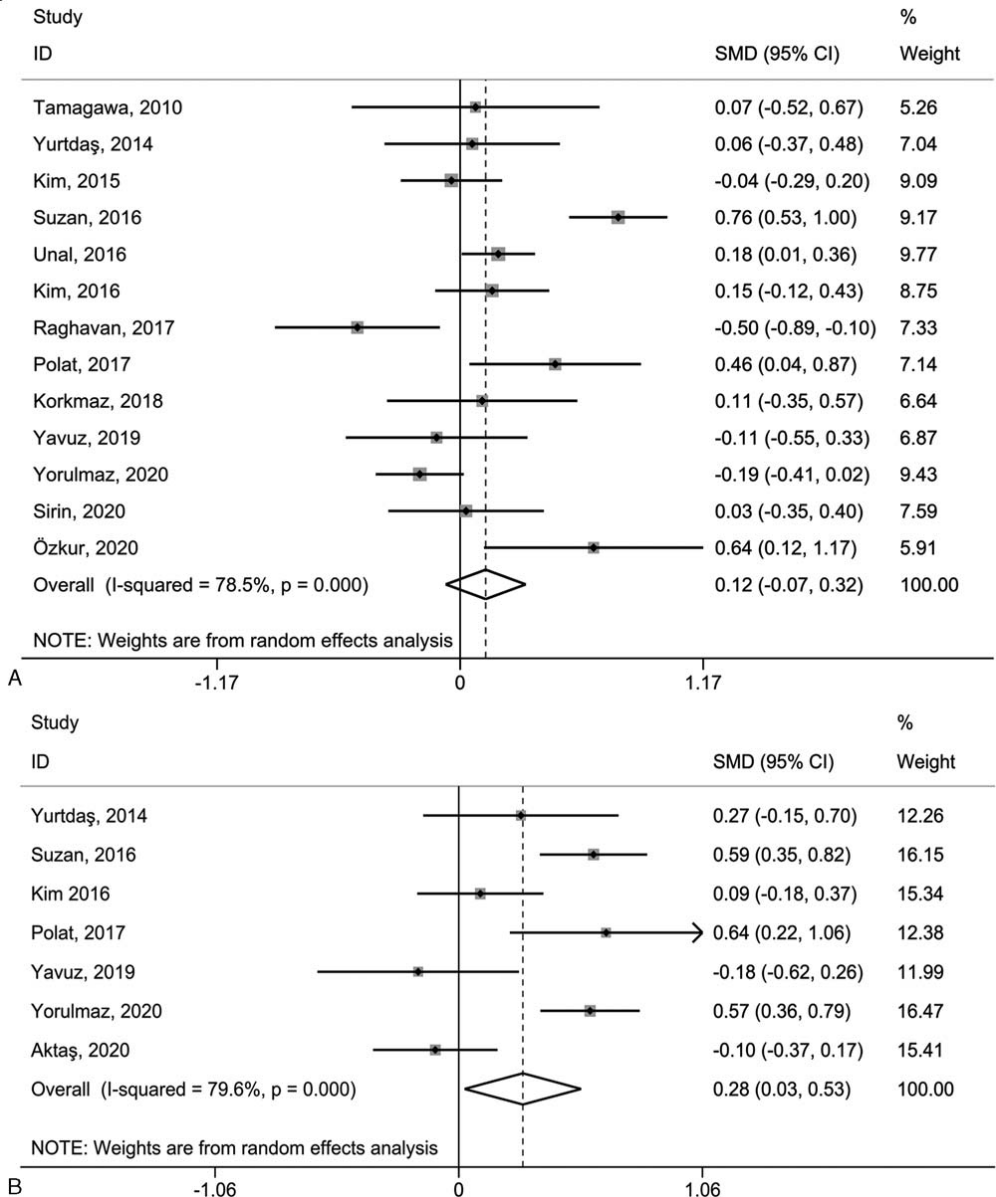
Forest plot for the relationship between PLT (A), and PLR (B) in psoriatic cases and normal controls. *P* values refer to the significance of heterogeneity; CI = confidence interval, PLR = platelet-to-lymphocyte ratio, PLT = platelet count, SMD = standardized mean difference.

**Figure 3:**
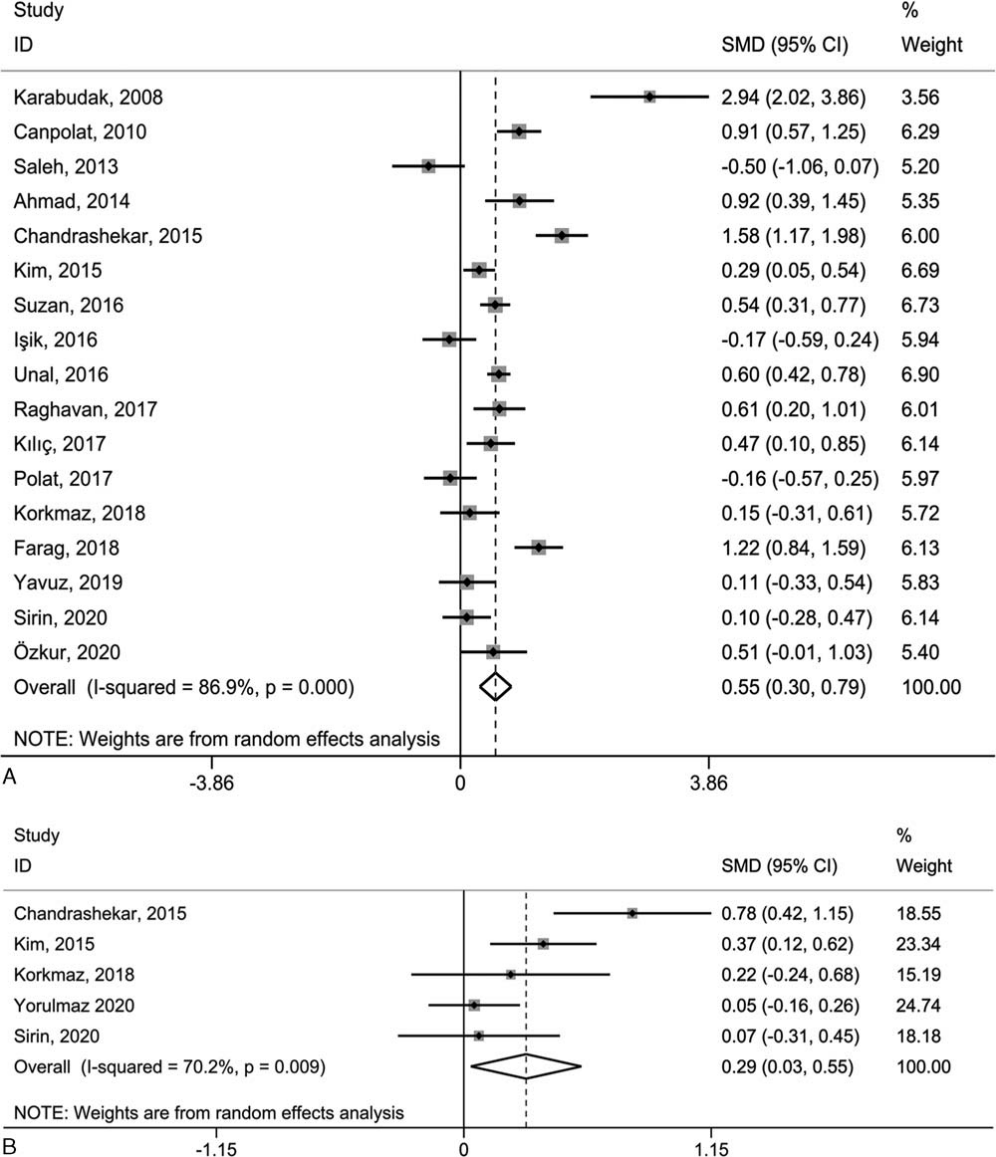
Forest plot for the relationship between MPV (A), and PDW (B) in psoriatic patients and normal individuals. *P* values refer to the significance of heterogeneity; CI = confidence interval, MPV = mean platelet volume, PDW = platelet distribution width, SMD = standardized mean difference.

To compare platelet-associated parameters between psoriatic patients and healthy controls, a REM meta-analysis using SMD with 95% CI was conducted. Specifically, PLT presented non-significant differences between psoriatic patients and normal individuals (SMD = 0.12, 95% CI = −0.07 to 0.32, *P* = .210, Fig. 2A), while PLR (SMD = 0.28, 95% CI = 0.03–0.53, *P* = .031, Fig. 2B), MPV (SMD = 0.55, 95% CI = 0.30–0.79, *P* < .001, Fig. 3A), and PDW (SMD = 0.29, 95% CI = 0.03–0.55, *P* = .027, Fig. 3B) were remarkably greater in psoriatic patients than in normal individuals. Significant heterogeneity between studies was measured in every contrast, with *I*^2^ scores between 70.2% and 86.9%.

### 3.5 Subgroup analysis

**Figure 4:**
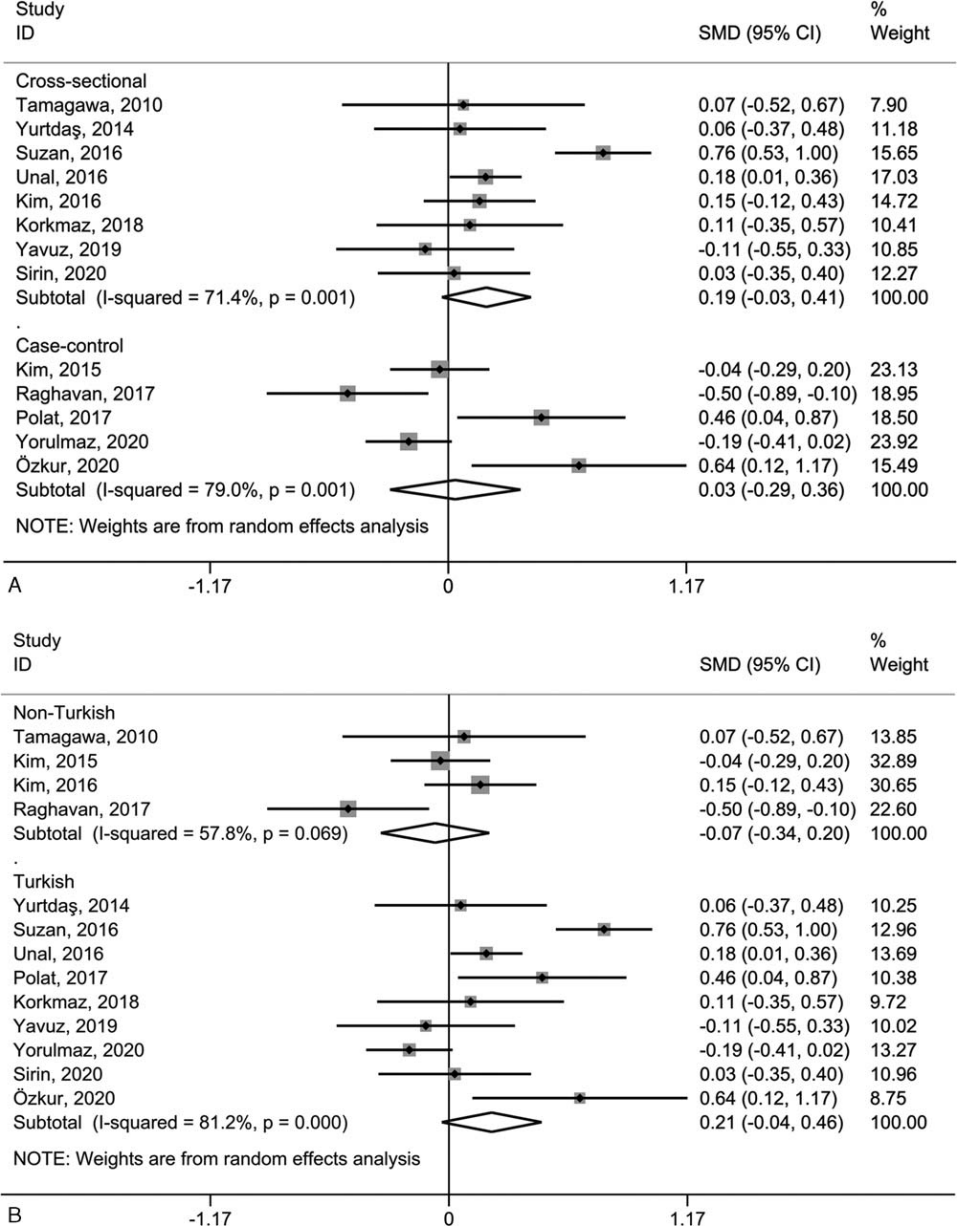
Subgroup studies of the included studies on PLT based on research design (A) and research regions (B) in the contrast between the psoriatic cases and normal individuals. “Cross-sectional” and “Case-control” refer to study design; *P* values refer to the significance of heterogeneity; CI = confidence interval, PLT = platelet count, SMD = standardized mean difference.

**Figure 5:**
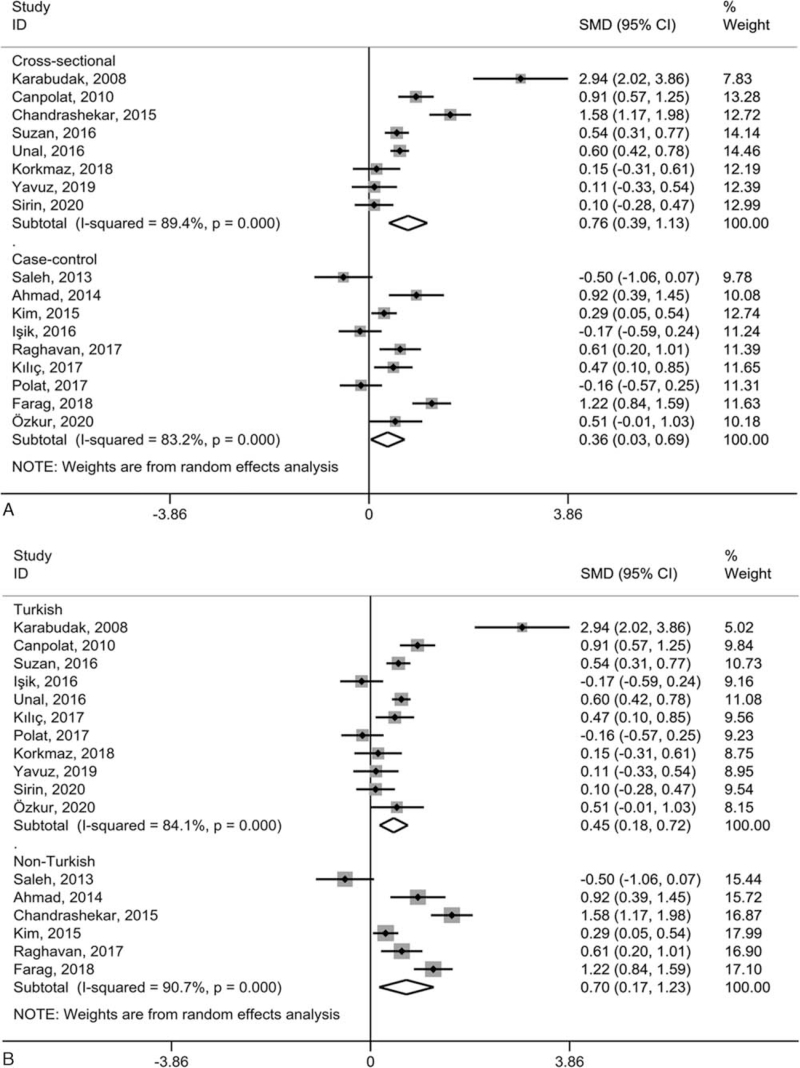
Subgroup studies of the included studies on MPV based on research design (A) and research regions (B) in the contrast between the psoriatic cases and normal individuals. “Cross-sectional” and “Case-control” refer to study design; *P* values refer to the significance of heterogeneity; CI = confidence interval, MPV = mean platelet volume, SMD = standardized mean difference.

Owing to the small number of included studies on PLR and PDW, subgroup analysis based on study design and study location was merely carried out in studies on PLT and MPV in the comparison between psoriatic patients and healthy individuals. Consistent with the overall results, PLT presented non-significant differences in subgroups stratified based on research design (Fig. 4A) and research regions (Fig. 4B). MPV was likewise consistent with the overall results that it was significantly higher in subgroups stratified according to the study design (Fig. 5A) and study location (Fig. 5B). However, heterogeneity remained high in all subgroup analyses.

### 3.6 Meta-regression analysis

To identify whether the potential moderating variables had impacts on the gathered effect size, a REM meta-regression study was performed with pre-defined independent variables reported in greater than or equal to 10 studies. We found that PLT was significantly associated with male proportion (*P* = .006), but not with age, sample size, the PASI score of patients, and the publishing time of selected studies (*P* > .05). MPV was significantly associated with publication year of the included studies (*P* = .034), but not with age, male proportion, sample size, disease duration, and the PASI score of patients (*P* > .05). The results are presented in more detail in Table 3.

**Table 3:** Meta-regression analysis demonstrating the association of platelet count and mean platelet volume with pre-established variables in psoriatic patients.

Variables	*Exp(B)*	*t*	95% confidence interval	*P*
Platelet count				
Publication year	0.979	−0.51	0.894–1.072	.618
Age	0.986	−0.57	0.934–1.041	.579
Male proportion	0.972	−3.48	0.954–0.990	**.006**
Sample size	1.000	0.280	0.998–1.003	.785
PASI score	0.968	−1.16	0.907–1.032	.275
Mean platelet volume				
Publication year	0.879	−2.33	0.782–0.989	**.034**
Age	0.963	−1.12	0.896–1.035	.280
Male proportion	0.998	−0.14	0.965–1.035	.967
Sample size	0.999	−0.13	0.995–1.005	.896
Disease duration	1.032	0.53	0.900–1.183	.609
PASI score	1.003	0.06	0.908–1.107	.953

PASI = the Psoriasis Area and Severity Index.

### 3.7 Sensitivity analysis

**Figure 6:**
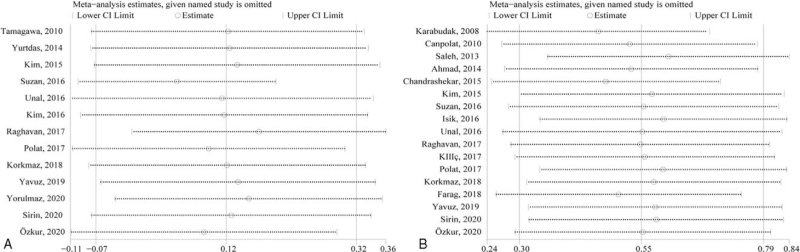
Sensitivity analysis for assessing the impact of every research on the combined PLT (A) and MPV (B) for the association between psoriatic cases and healthy controls. CI = confidence interval, MPV = mean platelet volume, PLT = platelet count.

Sensitivity analysis revealed that no single study had a huge impact on the pooled PLT (Fig. 6A) and MPV (Fig. 6B) between psoriatic patients and normal individuals.

### 3.8 Publication bias

**Figure 7:**
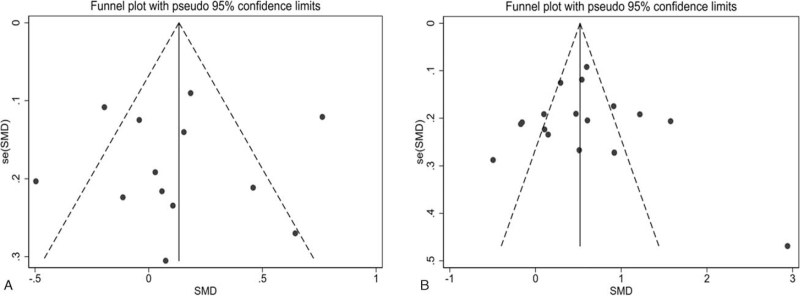
Observation of funnel plots assessing underlying publication bias of the selected researches on PLT (A) and MPV (B) for the association between psoriatic patients and healthy controls. se = standard error, MPV = mean platelet volume, PLT = platelet count, SMD = standardized mean difference.

We did not observe any evidence of publication bias in the combined survey of PLT (Fig. 7A) and MPV (Fig. 7B), which was verified by the Egger test (*P* = .880 and *P* = .763, respectively).

## 4 Discussion

The present study is the first meta-analysis of the association between platelet-associated parameters and psoriasis. The results of this study indicated that higher PLR, MPV, and PDW, but not PLT, are associated with psoriasis. Subgroup analysis showed that the pooled results were not affected by the study design and location. No obvious publication bias was observed among the included studies. Sensitivity analysis revealed that the pooled results were not influenced by any individual study. Therefore, the results of the present meta-analysis were stable and robust.

Platelet-associated parameters are easily available in routine laboratories with considerably low cost, high efficiency, and high reproducibility; therefore, they have long been considered a powerful tool for efficient diagnosis and therapeutic monitoring of multiple medical conditions. PLR is usually used as an indicator of chronic low-grade inflammation^[14]^; therefore higher PLR in psoriatic patients suggests that psoriasis is accompanied by systemic inflammation. As already mentioned, an increased PLR may be due to an elevated platelet number, decreased lymphocyte number, or both. In this meta-analysis, there was a trend toward higher PLT in psoriatic patients than in healthy controls, although the difference was not statistically significant. Therefore, a higher PLR in psoriatic patients, in part, may result from the increased PLT. Recently, a meta-analysis carried out by Paliogiannis et al^[39]^ evaluated the relationship between PLR and psoriasis, which was consistent with our findings that psoriatic patients presented a significantly higher PLR than healthy individuals; the current meta-analysis included 7 studies on PLR in the comparison between psoriatic patients and normal individuals, while Paliogiannis et al involved only 4 studies. Therefore, this study provides updated evidence of a significant association between PLR and psoriasis.

MPV is the most frequently used platelet parameter that reflects platelet activation in inflammatory and immune-related diseases.^[40]^ In this meta-analysis of 22 included studies, 17 examined MPV in psoriatic patients, which provided us with an adequate number of studies to conclude that patients with psoriasis are accompanied by increased MPV. Several previous meta-analyses also demonstrated that MPV was significantly higher in patients with metabolic diseases, such as diabetes, non-alcoholic fatty liver disease, and cardiovascular diseases.^[41–43]^ Currently, there is a growing consensus that psoriasis is a systemic disease that is intimately related to comorbid diseases such as diabetes, obesity, and cardiovascular diseases, although it is mostly present with local inflammatory lesions in the skin.^[44]^ Two recent meta-analyses consistently showed that lipid metabolism was abnormal in psoriatic patients, and there were significant associations between psoriasis and obesity and cardiovascular disease.^[45,46]^ Therefore, platelet activation may serve as a bridge between psoriasis and related metabolic diseases. Moreover, several studies included in this meta-analysis showed that plasma levels of platelet-derived microparticles, soluble P-selectin, and CD62, which closely reflect platelet activation, were also significantly elevated in patients with psoriasis.

Recently, increasing attention has been given to the relationship between PDW and a variety of diseases.^[47]^ PDW has usually been used to evaluate the volume heterogeneity of platelets, and an increased PDW indicates that the volume of platelets is not homogeneous.^[48]^ There is evidence indicating that PDW can more sensitively reflect the changes in platelet volume related to the formation of pseudopodia and shape changes during platelet activation than MPV.^[49]^ Therefore, a higher PDW is considered a sensitive indicator of platelet activation. Similar to MPV, recent studies have also suggested that PDW is positively associated with a higher risk of diabetes and cardiovascular diseases.^[50,51]^ However, among the 22 studies included in the present meta-analysis, only 5 reported the results of PDW. Therefore, more research is needed to substantiate the observation that there are significant associations between psoriasis and PDW.

This meta-analysis had several potential limitations. First, considerable heterogeneity was observed in all pooled outcomes. Although subgroup analysis suggested that the pooled results of PLT and MPV were not moderated by study design and study location, the sources of heterogeneity still cannot be reasonably explained. In the following univariate meta-regression analysis, significant negative correlations between PLT and the proportion of male patients and between MPV and publication year suggest that these 2 variables, in part, contribute to the heterogeneity in the pooled results of PLT and MPV. Meta-regression analysis can not only explore potential sources of heterogeneity, but also identify the relationships between the independent and dependent variables. In this study, we failed to find significant relationships between PASI score and PLT and MPV by meta-regression analysis, suggesting that both PLT and MPV cannot reflect the severity of psoriasis. Second, since the number of included studies on PLR and PDW was relatively small, we did not conduct subgroup and meta-regression analyses to explore the sources of heterogeneity, and we failed to evaluate whether PLR and PDW can reflect the severity of psoriasis. Moreover, some potential confounders such as the levels of inflammation and the use of drugs in patients are limited in the eligible studies, which prevented us from analyzing whether these factors contributed to the heterogeneity between studies. Third, psoriasis is a chronic inflammatory dermatosis, and platelet activation is involved in inflammatory processes. However, other than PLR, almost no other inflammatory parameters such as C-reactive protein and certain inflammatory cytokines, especially IL-1 and TGF-β, which have been demonstrated to be most strongly associated with platelet activation,^[52,53]^ were examined in the studies included in this meta-analysis. Therefore, we failed to identify the relationship between platelet-associated parameters and inflammatory markers in patients with psoriasis. Fourth, although the findings of our analyses are based on the merged results from multiple previously reported studies that can increase sample size to a certain extent, the overall sample size is still not large enough, especially for the analysis of PLR and PDW. Fifth, as nearly half of the studies did not provide information on blood analyzers for measuring platelet-associated parameters, we failed to carry out subgroup analysis according to different analyzers. Imperfect detection from earlier hematology analyzers may misidentify small red cells as platelets, resulting in falsely elevated PLT and MPV to varying degrees, which may be one of the main reasons for the significant negative correlation between MPV and publication year. Sixth, since over half of the studies included in this meta-analysis were conducted in Turkey (14 out of 22), the results of these meta-analyses may not be representative of the overall population. However, our subgroup analyses based on research regions showed that the pooled outcomes of both Turkish and non-Turkish cases were consistent with the overall results. Finally, selective bias was probably inevitable, as only articles published in English were selected for this meta-analysis.

## 5 Conclusions

In conclusion, this meta-analysis indicated that psoriatic patients had higher PLR, MPV, and PDW, rather than PLT, than healthy individuals. However, this study failed to find a relationship between platelet-associated parameters and psoriasis severity. Further studies are needed to confirm the current outcomes, especially the relationship between PLR, PDW, and psoriasis.

## Author contributions

**Conceptualization:** Zhongwei Zhou.

**Data curation:** Li Li, Jianxiu Yu.

**Writing – original draft:** Li Li, Jianxiu Yu.

**Writing – review & editing:** Zhongwei Zhou.
